# Designing a highly efficient type III polyketide whole-cell catalyst with minimized byproduct formation

**DOI:** 10.1186/s13068-024-02545-x

**Published:** 2024-07-03

**Authors:** La Xiang, Xuanxuan Zhang, Yanyan Lei, Jieyuan Wu, Guangru Yan, Wei Chen, Shizhong Li, Wenzhao Wang, Jian-Ming Jin, Chaoning Liang, Shuang-Yan Tang

**Affiliations:** 1 CAS Key Laboratory of Microbial Physiological and Metabolic Engineering, State Key Laboratory of Microbial Resources, Institute of Microbiology, Chinese Academy of Sciences, Beijing, 100101 China; 2 Beijing Key Laboratory of Plant Resources Research and Development, Beijing Technology and Business University, Beijing, 100048 China; 3 State Key Laboratory of Transducer Technology, Institute of Microbiology, Chinese Academy of Sciences, Beijing, 100101 China; 4 University of Chinese Academy of Sciences, Beijing, 100049 China; 5 State Key Laboratory of Mycology, Institute of Microbiology, Chinese Academy of Sciences, Beijing, 100101 China

**Keywords:** Chalcone synthase, Byproduct, Growth selection, Naringenin, Type III polyketide

## Abstract

**Background:**

Polyketide synthases (PKSs) are classified into three types based on their enzyme structures. Among them, type III PKSs, catalyzing the iterative condensation of malonyl-coenzyme A (CoA) with a CoA-linked starter molecule, are important synthases of valuable natural products. However, low efficiency and byproducts formation often limit their applications in recombinant overproduction.

**Results:**

Herein, a rapid growth selection system is designed based on the accumulation and derepression of toxic acyl-CoA starter molecule intermediate products, which could be potentially applicable to most type III polyketides biosynthesis. This approach is validated by engineering both chalcone synthases (CHS) and host cell genome, to improve naringenin productions in *Escherichia coli*. From directed evolution of key enzyme CHS, beneficial mutant with ~ threefold improvement in capability of naringenin biosynthesis was selected and characterized. From directed genome evolution, effect of thioesterases on CHS catalysis is first discovered, expanding our understanding of byproduct formation mechanism in type III PKSs. Taken together, a whole-cell catalyst producing 1082 mg L^−1^ naringenin in flask with* E* value (evaluating product specificity) improved from 50.1% to 96.7% is obtained.

**Conclusions:**

The growth selection system has greatly contributed to both enhanced activity and discovery of byproduct formation mechanism in CHS. This research provides new insights in the catalytic mechanisms of CHS and sheds light on engineering highly efficient heterologous bio-factories to produce naringenin, and potentially more high-value type III polyketides, with minimized byproducts formation.

**Supplementary Information:**

The online version contains supplementary material available at 10.1186/s13068-024-02545-x.

## Background

Polyketide synthases (PKSs) are classified into three types based on their enzyme structures [1]. Type III PKSs are ubiquitous in plants, bacteria, and fungi [2–4]. As an important class of enzymes, they synthesize a variety of commonly used and valuable natural aromatic products (secondary metabolites), including curcuminoid, chalcone, stilbene, coumarin, and chromone [5, 6]. A type III PKS, chalcone synthase (CHS), is also the key enzyme involved in flavonoids biosynthesis [7, 8]. Type III PKSs generally catalyze the iterative condensation of malonyl-coenzyme A (CoA) with a CoA-linked starter molecule. They use free CoA thioesters as substrates, in contrast to type I/II PKSs, which employ acyl carrier proteins for catalysis. Type III PKSs also have broad substrate specificities, making them ideal for engineering the synthesis of diverse polyketide compounds [9]. However, despite their utility and simpler structures, our understanding of type III PKSs lags far behind that of type I/II PKSs.

Type III PKSs catalyze the formation of many valuable secondary metabolites; nevertheless, their catalytic efficiencies are limited and these products usually exist in low amounts in nature. Previous studies have been focused on improving the diversity of secondary metabolites produced, based on the broad source and substrate specificity of type III PKSs [6, 10]. Engineering strategies have also been applied to broaden the function diversity of type III PKSs to obtain various polyketide compounds [9]. The catalytic efficiency of these enzymes is critical for the recombinant overproduction of valuable secondary metabolites in microbial cell factories; however, there are few reports on type III PKSs engineering for improved catalytic activities [11], likely due to the hardly simulated intracellular environments in whole-cell biosynthesis (e.g., the low and dynamically variable concentrations of CoA precursors). Beneficial enzyme mutants obtained from in vitro engineering frequently perform poorly during in vivo biosynthesis because of the different work environment. Thus, in vivo-directed evolution under real background metabolic conditions is preferred for type III PKS biosynthesis, yet a lack of rapid screening methods retards the application of this strategy.

**Fig. 1:**
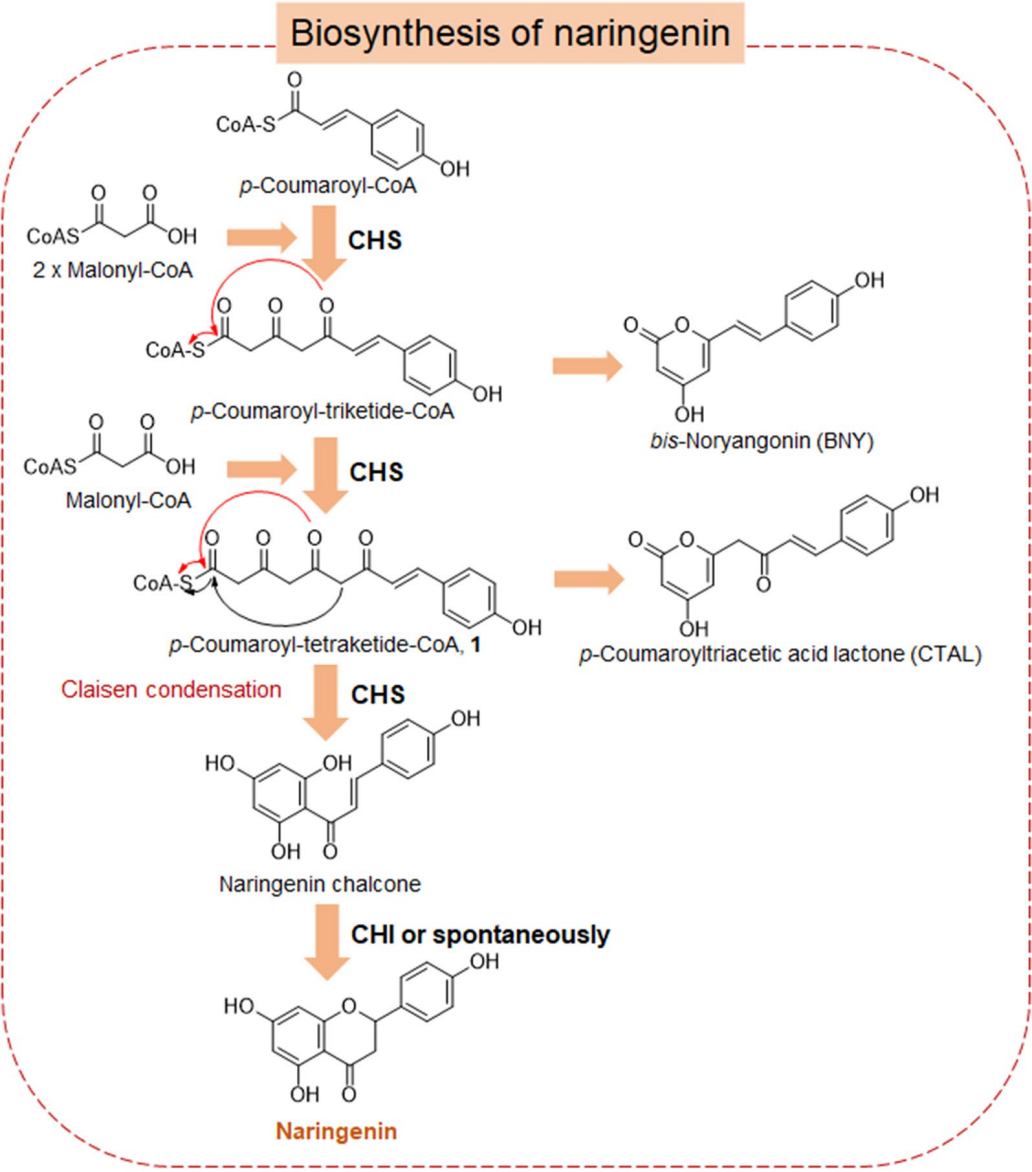
Biosynthesis of naringenin. CHS, chalcone synthase; CHI, chalcone isomerase; CHIL, CHI-like protein; compound **1** is catalytic intermediate

CHS is the most well studied among type III PKSs [12–14]. It catalyzes the condensation of one molecule of *p*-coumaroyl-CoA and three molecules of malonyl-CoA to form naringenin chalcone, which is converted to naringenin, either spontaneously or by chalcone isomerase (CHI) [8] (Fig. 1). Variation in the volume of CHS active sites has been shown to alter the size of acceptable starter units and result in the formation of different final polyketide products [13, 15]. Apart from chalcone products, CHS has been shown to produce the derailment byproducts *p*-coumaroyltriacetic acid lactone (CTAL) [16, 17] and *bis*-noryangonin (BNY) [18] via the lactonization of reaction intermediates (Fig. 1). So far no way has been reported to reduce byproducts formation, resulting in low naringenin chalcone yields. Besides CHS, byproducts formation frequently occurs in most other type III PKSs catalysis [18–20], greatly reducing the biosynthesis of desired products. Recently, a CHI-like protein (CHIL) was reported to interact with CHS and enhance naringenin chalcone biosynthesis while reducing CTAL formation [21, 22], thereby facilitating the flux of carbon from substrates to the flavonoid pathway. However, the mechanisms underlying this process remain unclear.

Cell growth-based screening methods are usually simple and rapid, preferable for in vivo-directed evolution [23, 24]. In this study, a growth selection system was developed for directed evolution of in vivo biosynthesis of naringenin chalcone, the precursor of naringenin. Beneficial CHS mutants have been obtained. In further directed genome engineering, thioesterases was discovered to impact CHS catalysis. These findings expand our understanding of the mechanism of byproduct formation in type III PKS catalysis. A whole-cell catalyst of naringenin with both enhanced efficiency and significantly reduced byproduct formation was then designed based on our results.

## Methods

### General

Restriction enzymes and DNA polymerase were purchased from Takara (Shiga, Japan). Gibson Assembly Kit was purchased from New England Biolabs (MA, USA). Primers were synthesized by Life Technologies (Shanghai, China). Naringenin chalcone, naringenin, *p*-coumaric acid, malonyl-CoA and *p*-coumaroyl-CoA were purchased from Sigma-Aldrich (St. Louis, USA). CTAL was prepared using preparative high-performance liquid chromatography (HPLC) and deduced on basis of ESI Mass which showed by a quasi-molecular ion peak at *m/z* 273 [M + H] (Fig. S1).

*E. coli* MC1061 was used for plasmid construction. Strain CUR01 [25] was used for library screening and product biosynthesis of CHS. All bacteria were routinely grown in Luria–Bertani (LB) medium (10 g L^−1^ tryptone, 5 g L^−1^ yeast extract, 5 g L^−1^ NaCl). The antibiotics ampicillin (100 μg mL^−1^) and kanamycin (50 μg mL^−1^) were used when necessary. Library screening and product biosynthesis were done in yeast extract M9 media (YM9, containing 1 × M9 salts, 10 g L^−1^ yeast extract, 3% glycerol, and 42 g L^−1^ MOPS [3-(N-morpholino) propanesulfonic acid]).

### Plasmid construction

The plasmids and strains used in this study are listed in Table S1. The primers used in this study are listed in Table S2.

*pTrc99a-4CL2M/4AT* Gene encoding *p*-coumarate:CoA ligases 4CL2 (GenBank accession No. 821678) was amplified with primers 4CL2-G-For and 4CL2-G-Rev using *Arabidopsis thaliana* cDNA as template. The PCR products were assembled with the fragment amplified with primers pTrc99a-G-For and pTrc99a-G-Rev using plasmid pTrc99a (Sangon, China) as template. Then amino acid substitutions M293P and K320L [26] were introduced in 4CL2 via site-directed mutagenesis, resulting in plasmid pTrc99a-4CL2M. Similarly, gene encoding 4AT (a mutant of 4CL1 from *A. thaliana*) was amplified with primers 4AT-G-For and 4AT-G-Rev using plasmid pGAP-CHS-4CL [20] as template. The PCR products were assembled with above-mentioned pTrc99a fragment, resulting in plasmid pTrc99a-4AT.

*pYk-CHS* Primers CHS-pY-For and CHS-pY-Rev were used to amplify the fragment containing gene encoding CHS from *Petunia hybrida* using plasmid pGAP-CHS-4CL [20] as template, and the fragment was assembled with the PCR product amplified with primers pY-CHS-G-For and pY-CHS-G-Rev using plasmid pYk-CUS M1 [25] as template, resulting in plasmid pYk-CHS.

*pET28a-CHS* Primers CHS-G-For and CHS-G-Rev were used to amplify the fragment containing gene encoding CHS using plasmid pYk-CHS as template, and the fragment was assembled with the PCR product amplified with primers 28a-CHS-G-For and 28a-CHS-G-Rev using plasmid pET28a as template, resulting in plasmid pET28a-CHS.

*pET28a-TesB* The *tesB* gene was amplified with primers tesB-GB-For and tesB-GB-Rev using genomic DNA of strain CUR01 as template. The PCR product was assembled with the PCR product amplified with primers pET-tesB-For and pET-tesB-Rev using plasmid pET28a as template, resulting in plasmid pET28a-TesB.

*pET28a-CHIL* Primers CHIL-For and CHIL-Rev were used to amplify the *CHIL* gene from *A. thaliana* (GenBank accession No. 830409) synthesized by General Biosystems (Anhui, China). The PCR product was assembled with the vector fragment amplified with primers pET28a-CHIL-For and pET28a-CHIL-Rev using pET28a as template, resulting in plasmid pET28a-CHIL.

*pTrc99a-4CL2M-CHIL* Using pET28a-CHIL as template, gene *CHIL* fragment was amplified with primers CHIL-gibson-For and CHIL-gibson-Rev. The PCR products were assembled with the vector fragment amplified with primers 4CL2M-gibson-For and 4CL2M-gibson-Rev using pTrc99a-4CL2M as template, using Gibson Assembly Kit, resulting in plasmid pTrc99a-4CL2M-CHIL.

### Strain construction

Five thioesterase genes, *tesB*, *yigI*, *yciA*, *fadM* and *paaI*, were sequentially knocked out in strain CUR01 [25] with λ-Red recombination method. The FRT-flanked kanamycin resistance gene was eliminated using flippase-mediated recombination as described [27], resulting in strain NAR01.

### Construction of the random mutagenesis library

The random mutagenesis libraries of CHS were constructed with error-prone PCR. Primer pairs CHS-EP-For/CHS-EP-Rev were used to amplify the *chs* gene, using plasmid pYk-CHS as templates. The PCR reaction mixture consisted of 5 mM MgCl_2_, 0.2 mM each of dATP and dGTP, 1 mM each of dCTP and dTTP, 0.1 mM MnCl_2_ and rTaq DNA polymerase. Then the PCR products obtained, containing randomly mutated *chs* gene, were used as megaprimer to perform MEGAWHOP PCR using plasmid pYk-CHS as template. Following the MEGAWHOP PCR, *Dpn*I digestion (20 U) of the template was performed at 37 °C for 2 h. The *Dpn*I was inactivated at 80 °C for 20 min. Then the PCR products were used to transform *E. coli* MC1061 strain and around 10^6^ transformants were recovered. Ten randomly picked clones from the library were sequenced and contained an average of 3 nucleotide mutations per kilobase pairs. All colonies from the agar plates were used for plasmid isolation to prepare the plasmid library.

### Screening of the mutagenesis libraries

The random mutagenesis library of CHS was used to transform strain CUR01 harboring plasmid pTrc99a-4CL2M. The transformants were cultured in YM9 medium at 37 °C until OD_600_ reached 0.6, then 1 mM l-arabinose, 0.4 mM isopropyl β-d-1-thiogalactopyranoside (IPTG) and 4.5 mM *p*-coumaric acid (for CHS library) were added and the cells were allowed to grow at 30 °C for another ten hours. The cells were collected and used for the next round of screening. The screening was repeated for six rounds. Finally, cells were grown at 30 °C for 24 h on YM9 agar plates supplemented with 1 mM l-arabinose, 0.4 mM IPTG and 4.5 mM *p*-coumaric acid. The largest clones, representing mutants converting more *p*-coumaroyl-CoA than the parental enzyme, were selected and cultured in shake flasks to determine naringenin productions.

### Site-saturation mutagenesis

For site-saturation mutagenesis at T131 position of CHS, PCR was performed with primers CHS-T131-SM-For and CHS-EP-Rev, using plasmid pYk-CHS as template. Then the PCR product was used as megaprimer to perform MEGAWHOP PCR using plasmid pYk-CHS as template. Following the MEGAWHOP PCR, *Dpn*I digestion (20 U) of the template was performed at 37 °C for 2 h. The *Dpn*I was inactivated at 80 °C for 20 min. Then the PCR products were transformed into *E. coli* MC1061, resulting in plasmids pYk-CHS containing the saturated mutations at 131 position.

### HPLC quantification

For naringenin and CTAL quantification, a colony of strain CUR01 or NAR01 harboring plasmid pairs pTrc99a-4CL2M/pYk-CHS or pTrc99a-4CL2M-CHIL/pYk-CHS was inoculated in YM9 and cultured at 37 °C until OD_600_ reached 0.6, then 1 mM l-‍arabinose, 0.4 mM IPTG and 4.5 mM *p*-coumaric acid were added. The cells were further cultured at 30 °C for 48 h. A volume of 500 μL culture broth was mixed with 500 μL ethanol. After vortexing and centrifugation, the supernatant was used for HPLC analysis.

Quantification was performed with the Shimadzu LC-20AT system (Shimadzu Corporation, Kyoto, Japan) and a Waters symmetry C18 column (5 μm, 250 mm × 4.6 mm) working at 35 °C. Mobile phase A was 0.1‰ formic acid, and B was acetonitrile. A linear gradient of mobile phase B (5–30 min, 30–50%) with a flow rate of 0.5 mL min^−1^ was used for separation. Naringenin, CTAL, *p*-coumaric acid, *p*-coumaroyl-CoA and naringenin chalcone were detected at 287, 320, 305, 365 and 315 nm, respectively. The concentrations were calculated from the standard curves prepared with corresponding authentic compounds.

### Enzyme purification

A colony of BL21(DE3) harboring plasmid pET28a-CHS expressing CHS wild-type/mutant, or plasmid pET28a-TesB, or plasmid pET28a-CHIL, was grown in LB medium at 37 °C and induced with 0.4 mM IPTG when OD_600_ reached 0.6, then the cells were continuously grown at 30 °C for 12 h. Cells were harvested by centrifugation and resuspended in 100 mM Tris–HCl buffer (pH 8.0, 300 mM NaCl and 10 mM imidazole). After sonication with a JY92-IIN Ultra Sonic Cell Crusher (Ningbo, China), cell debris were removed by centrifugation and the supernatants were loaded on a pre-equilibrated nickel–nitrilotriacetic acid (Ni–NTA) column (Qiagen, Valencia, USA). The column was washed with wash buffer (100 mM Tris–HCl, 300 mM NaCl and 20 mM imidazole, pH 8.0), and the bound protein was eluted with the elution buffer (100 mM Tris–HCl, 300 mM NaCl and 250 mM imidazole, pH 8.0). Imidazole was removed by dialysis at 4 °C against 100 mM potassium phosphate buffer (pH 8.0).

The purity of proteins was assessed by sodium dodecyl sulfate polyacrylamide gel electrophoresis (SDS-PAGE) and the protein concentrations were determined with Bradford method [28].

### Specific activity determination of CHS

The standard reaction mixture consisted of 100 mM HEPES–NaOH buffer (pH 7.5), 50 μM *p*-coumaroyl-CoA, 150 μM malonyl-CoA, and purified CHS in a final volume of 50 μL. The mixture without protein was pre-incubated at 30 °C for 5 min, and then the reaction was started by adding CHS. After 1 h incubation at 30 °C, the reaction was stopped by adding 50 μL ethanol. Then the reaction products were analyzed with HPLC. One unit (U) of CHS activity was defined as the amount of enzyme required to produce 1 nmol naringenin per min.

### Molecular docking

The compound **1** was docked with CHS wild-type and T131A, respectively, using YASARA. The molecular dynamics simulations of the complexes were performed using YASARA. The simulation system was placed in a cubic periodic cell with water molecules. The simulation environment was set at pH 7.5. After hydrogenation and energy minimization step, the system was gradually heated to 303.15 K in AMBER14 force filed, and a 20 ns production of molecular dynamics simulation was performed and saved snapshot every 10 ps.

The CHS–CHIL complex structure was performed using Alphafold *v2.0*. And the compound **1** was docked with the CHS–CHIL complex structure using YASARA. Two substrate-binding frames were defined based on the binding site of CHS and CHIL, respectively. And the protein-compound **1** complex structure were generated through simulation.

### In vitro enzyme reactions

The standard reaction mixture consisted of 100 mM HEPES–NaOH buffer (pH 7.5), 50 μM *p*-coumaroyl-CoA, 150 μM malonyl-CoA, and purified CHS (0.24 μM) in a final volume of 50 μL. Purified CHIL (2.4 μM) or TesB (0.8 μM) was added when necessary. The mixture without enzymes was pre-incubated at 30 °C for 5 min, and then the reaction was started by adding CHS. CHIL was added together with CHS, while TesB was added 30 min after the reaction started. After 2 h incubation at 30 °C, the reaction was stopped by adding 50 μL ethanol. Then the reaction products were analyzed with HPLC.

### Statistics

Error bars indicate standard deviations from three parallel experiments. All *P* values were generated from two-tailed t-tests using the Microsoft Excel 2016 (Microsoft Corporation, USA).

## Results and discussion

### A growth selection system for the rapid evolution of type III PKSs

Microbial biosynthesis is an efficient way to produce many valuable natural products derived from plants, however, most recombinant microbial production systems show low productivities owing to inefficient pathway enzymes. Improving the catalytic efficiency of these enzymes is closely related to the intracellular environment (e.g., enhancing the affinity for precursors with low intracellular concentrations or the capacity to use major cofactors in host cells). In vivo-directed evolution allows for the screening of biosynthetic capabilities in real metabolic environments and offers the greatest probability of obtaining the best-performing mutants for whole-cell biosynthesis [20, 29, 30]. High-throughput screening methods are critical for successful in vivo-directed evolution. Although biosensors for specific final products are important tools for in vivo high-throughput screening [30, 31], strategies that are readily adapted to the screening of enzyme groups with similar functions are more favorable. In this study, a growth selection system used for the in vivo-directed evolution of CHS was developed based on the inhibition of cell growth by *p*-coumaroyl-CoA starter molecule.

**Fig. 2:**
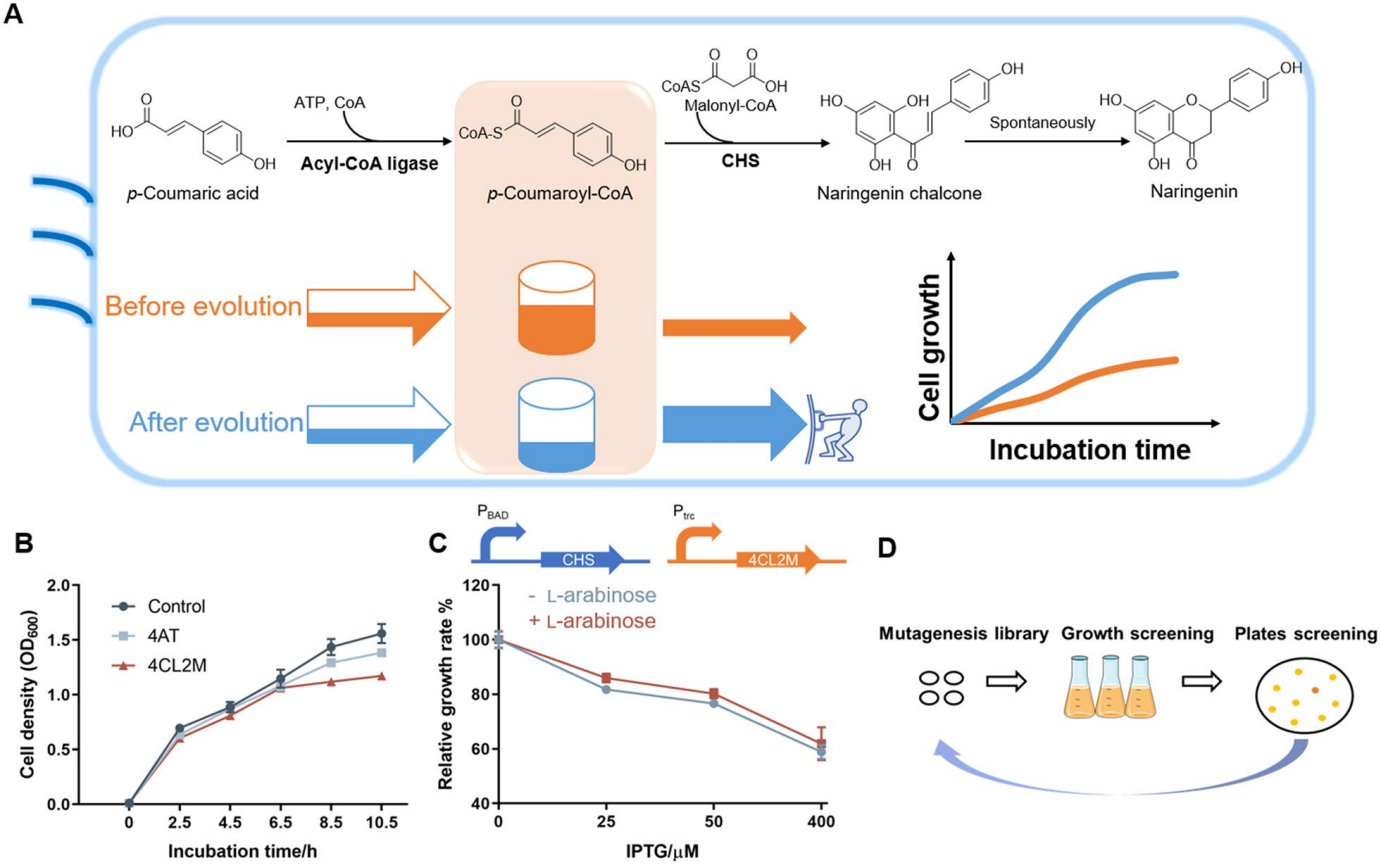
Development of a growth selection system for CHS engineering. **A** Scheme of in vivo biosynthetic pathway and the growth selection principle; **B** growth inhibition of cells overexpressing *p*-coumarate:CoA ligase 4AT and 4CL2M when *p*-coumaric acid (4.5 mM) was supplemented. Cells expressing 4CL2M in the absence of *p*-coumaric acid was used as control; **C** co-expression of CHS alleviated growth inhibition. Relative growth rate is calculated as the percentage of cell growth in the presence compared to in the absence of *p*-coumaric acid; **D** flowchart of in vivo-directed evolution of CHS

In type III polyketide biosynthesis, starter acyl-CoA substrates are usually synthesized by acyl-CoA ligases with corresponding carboxylic acids as substrates, using whole-cell catalysts (Fig. 2A). Strain CUR01 [25] (Table S1) was used for naringenin chalcone and naringenin biosynthesis in this study. Two *p*-coumarate:CoA ligases 4CL2M and 4AT were overexpressed in CUR01. Upon *p*-coumaric acid supplementation, cell growth was significantly inhibited, especially in the group of 4CL2M (Fig. 2B), which indicated that accumulation of excessive intracellular acyl-CoAs can be cytotoxic [32, 33] and disrupt CoA metabolism. Furthermore, a derepression in growth was observed with CHS co-expression. But the inhibition in cell growth did not completely recover, indicating that CHS activity was insufficient (Fig. 2C). Thus, the extent of cell growth recovery under substrate *p*-coumaric acid supplementation can be used to screen CHS activity. Through iterative rounds of cell growth screening, the cells harboring improved CHS which exhibited higher capabilities of *p*-coumaroyl-CoA conversion would be enriched. This growth selection mechanism can be potentially extended to the ultrahigh-throughput screening of most type III PKS activities (Fig. 2A). In addition, this selection system was simple, rapid and low-equipment dependent.

### CHS engineering

**Fig. 3:**
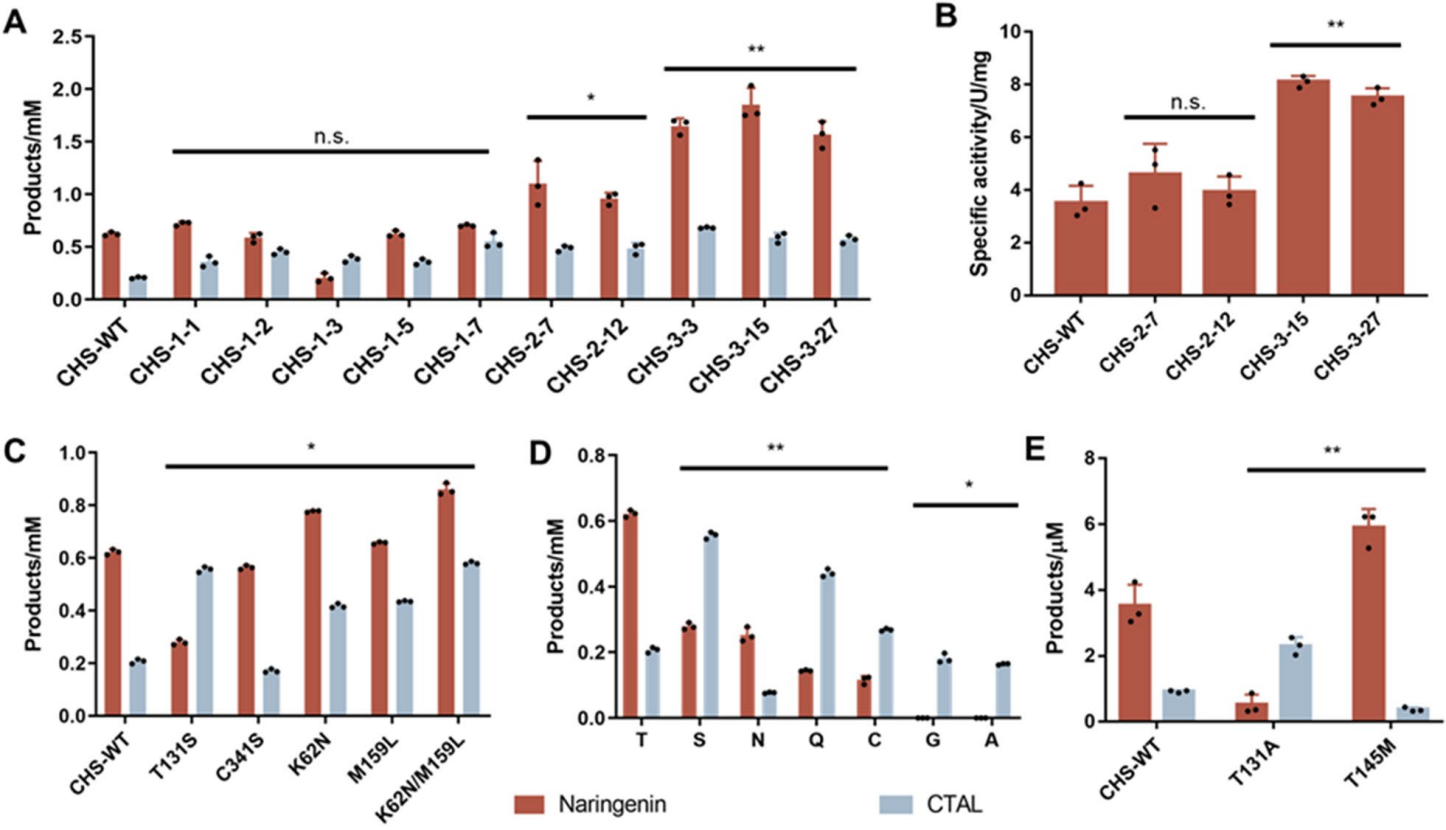
Engineering CHS for improved catalytic efficiency. **A** Naringenin and CTAL productions from strain CUR01 harboring the naringenin biosynthetic pathway expressing CHS mutants selected from three rounds of random mutagenesis; **B** specific activities of CHS mutants selected from the 2nd and 3rd rounds of random mutagenesis;** C** naringenin and CTAL productions from strain CUR01 harboring the indicated CHS mutants; **D** naringenin and CTAL productions from strain CUR01 harboring CHS with T131 saturated mutated. The mutants displaying no activity were not shown; **E** naringenin and CTAL productions by the indicated purified CHS enzymes, using *p*-coumaroyl-CoA and malonyl-CoA as substrates. CHS-WT, CHS wild-type. Results are shown as mean ± SD from at least n = 3 biological replicates in each experimental condition, n.s., not significant, *p < 0.05, **p < 0.005, Student’s two-tailed t-test

The growth selection system was then applied to engineer CHS in a naringenin biosynthetic pathway (Fig. 2D) that consisted of a 4CL2M enzyme and the CHS random mutagenesis library in strain CUR01, with 4.5 mM *p*-coumaric acid as substrate. After enrichment through five rounds of screening, strains from the library were mounted on agar plates, five largest clones were selected, and the products were analyzed using HPLC. As shown in Fig. 3A, in contrast to the wild-type CHS, mutants CHS-1-1, -2, -3, -5, and -7 exhibited improved production of naringenin or CTAL byproducts, whereas no naringenin chalcone was detected (Fig. S2). Sequencing results revealed the amino acid substitutions of these mutants (Table 1).

**Table 1:** Mutations of CHS mutants

CHS mutant	Mutation sites
CHS-1-1*	K57R, M159L, K281E
CHS-1-2	V98A, I279V
CHS-1-3	T131S, C341S
CHS-1-5	K62N, T194S, A195A (GCT-GCA), V232V (GTT-GTA)
CHS-1-7	K62N, M159L
CHS-2-7*	K57R, M159L, K281E, K9R, V100D, G334G (GGT-GGG)
CHS-2-12	K57R, M159L, K281E, L147Q, G200G (GGT-GGA), V261F
CHS-3-3	K57R, M159L, K281E, K9R, V100D, G334G (GGT-GGG), I309I (ATC-ATT)
CHS-3-15	K57R, M159L, K281E, K9R, V100D, G334G (GGT-GGG), L358Q
CHS-3-27	K57R, M159L, K281E, K9R, V100D, G334G (GGT-GGG), S297N

^*^indicated the selected mutants used as template for the next round of library generation and screening

Mutant CHS-1-1 was used as the parent enzyme to construct a library for the second round of random mutagenesis. Two mutants that produced higher levels of both naringenin and CTAL were selected in this round, and mutant CHS-2-7 was used as the parent enzyme for a third round of random mutagenesis, during which three mutants, CHS-3-3, -15, and -27, were obtained. Mutant CHS-3-15 displayed an approximately threefold increase in naringenin production relative to the wild-type enzyme, making it the best CHS mutant obtained in this study (Fig. 3A). The specific activities of the purified CHS-2-7, 2-12, 3-15, and 3-27 mutants were then determined. Since naringenin chalcone was rapidly converted to naringenin (Fig. S2), naringenin production in the presence of two acyl-CoA substrates was used to evaluate enzyme activity. Compared to the wild-type CHS, the activities of CHS-2-7, 2-12, 3-15, and 3-27 were increased by 1.31-, 1.12-, 2.30-, and 2.13-fold, respectively (Fig. 3B).

### Evaluation of byproduct formation by CHS mutants

Since the principle of our growth selection system was based on the consumption of the acyl-CoA starter molecule, it did not exclude mutants forming derailment byproducts, whereas elimination of byproducts formation and improvement of product specificity is of great necessity to increase biosynthesis of the desired product naringenin. *E* values [22] were introduced to evaluate the product specificities of the CHS whole-cell catalysts. Since naringenin chalcone was rapidly converted to naringenin and BNY (Fig. 1) was almost negligible, naringenin concentration [NAR] and CTAL concentration [CTAL] were used for *E* value calculation as follows:

$$E=\frac{\left[\text{NAR}\right]-[\text{CTAL}]}{\left[\text{NAR}\right]+[\text{CTAL}]}\times 100\text{\%}.$$

A positive *E* value inferred that the rate of naringenin production exceeded that of CTAL formation, whereas a negative *E* value inferred that the rate of CTAL formation exceeded that of naringenin production. It was found that *E* value of the best mutant CHS-3-15 was 50.1%, almost similar with that of wild-type enzyme (50.0%). Mutants from the first round of screening were found to produce high levels of CTAL byproduct (Fig. 3A). Among them, mutant CHS-1-3 exhibited a negative *E* value, indicating that the rate of CTAL formation exceeded that of naringenin chalcone production (Fig. S3A). To explore the contributions of two amino acid substitutions in mutant CHS-1-3 (Table 1) to CTAL formation, two single mutants—T131S and C341S—were constructed. In particular, T131S was proved critical to the reduction of naringenin and improved CTAL production (Fig. 3C and S3B). The T131 position is located in the substrate-binding pocket. Site-saturation mutagenesis of T131 was performed, and only six mutants showed detectable activity. Among them, substitutions S and Q produced less naringenin but much higher levels of CTAL than the wild-type enzyme. Both T131A and T131G produced almost no detectable naringenin but significant levels of CTAL, with *E* values close to − 100% (Fig. 3D and S3C). We further explored the product profile of purified T131A mutant in vitro. Similar to that in in vivo reactions, naringenin production was dramatically reduced, whereas high levels of CTAL were generated. Thus, this mutation significantly improved byproduct formation in vivo and in vitro (Fig. 3E).

Mutants CHS-1-1 and -7 shared an M159L mutation, whereas CHS-1–5 and -7 shared a K62N mutation. Thus, two mutants with single mutations—K62N and M159L—were constructed. Both of them were found to contribute to improvements in naringenin and CTAL production, and an additive effect was observed when both mutations occurred simultaneously (Fig. 3C).

### Host-cell engineering for improved naringenin synthesis

**Fig. 4:**
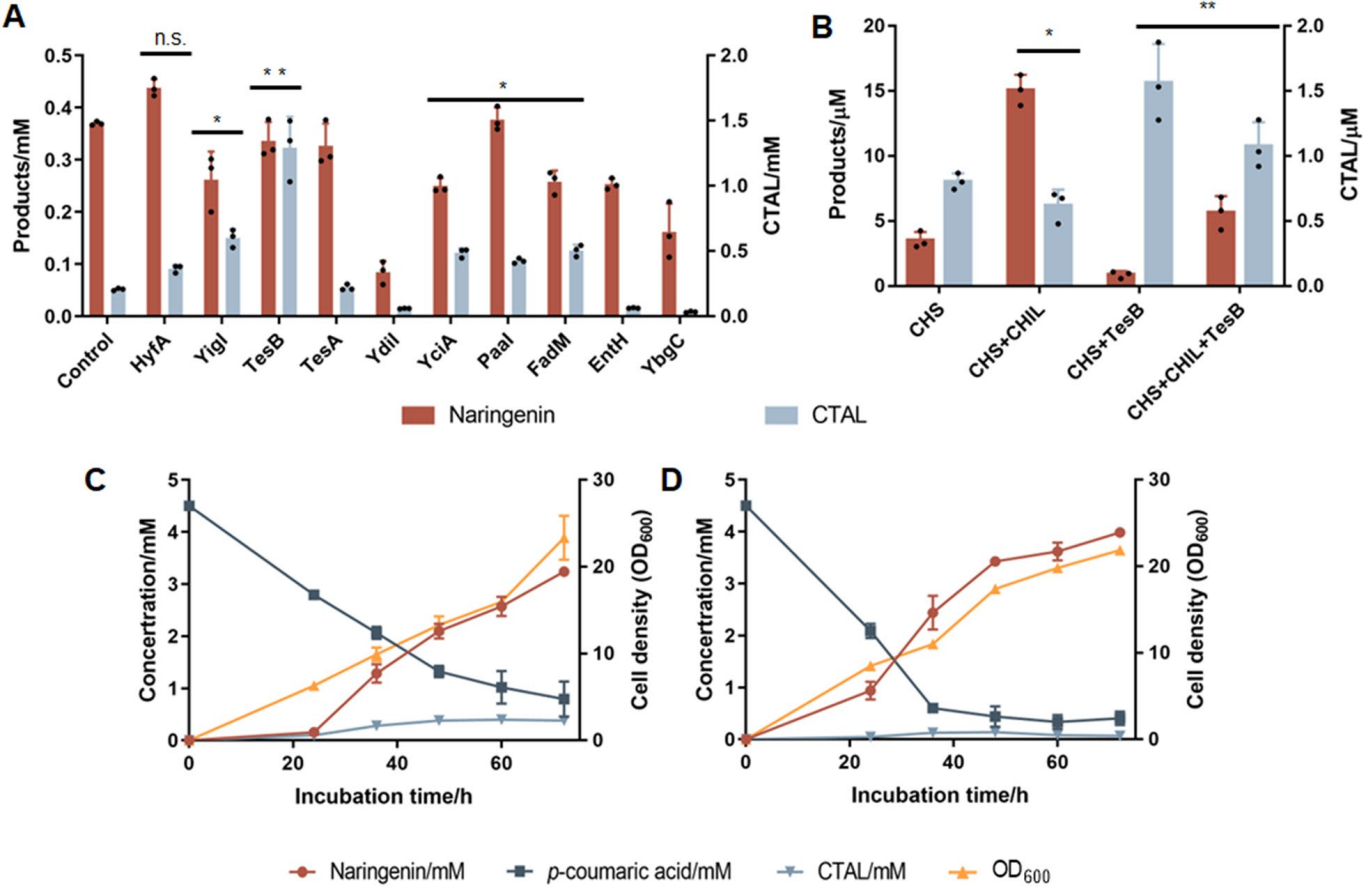
Directed genome engineering for improved naringenin synthesis.** A** Naringenin or CTAL productions of strain CUR01 harboring wild-type naringenin biosynthetic pathway co-expressed with the indicated proteins; **B** naringenin and CTAL productions of purified CHS in the presence of purified CHIL or TesB. Biosynthesis of naringenin by CHS3-15 mutant in strain NAR01, in the absence (**C**) or presence (**D**) of CHIL expression. The results are shown as mean ± SD from at least n = 3 biological replicates in each experimental condition, n.s., not significant, *p < 0.05, **p < 0.005, Student’s two-tailed t-test

As shown in Fig. 1, apart from type III PKSs, the availability of intracellular malonyl-CoA, CoA, and adenosine triphosphate (ATP) affected the biosynthetic efficiency of the final polyketide products. Therefore, the metabolic network of host cells plays an important role in type III polyketide biosynthesis, and the growth selection system could also be applied in host cell genome engineering. Gene targets that promote the conversion of acyl-CoA would improve the cell growth. The *E. coli* ASKA (A Complete Set of *E. coli* K-12 ORF Archive) [34] library was used to transform strain BW25113, which expressed the naringenin biosynthetic pathway, and the mutants with improved production were screened via our growth selection system. Three colonies were selected for naringenin and CTAL determination, although none of them contributed greatly to naringenin biosynthesis, the CTAL formations were improved. Sequencing results revealed that the overexpressed genes were *hyfA*, *yigI* and *tesB* (Table S3). Interestingly, both *yigI* and *tesB* encode thioesterases, and the overexpression of *tesB* resulted in a 6.23-fold increase in CTAL production (Fig. 4A). Gene *tesB* encodes a thioesterase II, which has a relatively broad substrate specificity and cleaves medium- and long-chain acyl-CoAs [35].

To further explore the effects of thioesterases on CHS activity, another seven genes encoding thioesterases in *E. coli* were individually selected for co-expression with the naringenin biosynthetic pathway. Notably, none of the thioesterases facilitated naringenin production, whereas three led to improved CTAL production (negative *E* values). Among all the tested thioesterases, the overexpression of TesB yielded the highest CTAL production, and the *E* value reached − 58.6% (Figs. 4A and S3D).

### Exploring the byproduct formation mechanism in CHS catalysis

Unexpectedly, some thioesterases significantly improved the biosynthesis of CTAL byproducts in CHS reactions. To remove the intracellular background, in vitro reactions of purified CHS in the presence or absence of purified TesB were performed, using *p*-coumaroyl-CoA and malonyl-CoA as substrates. We found that in the presence of TesB, CTAL formation was improved while naringenin synthesis declined (Fig. 4B). Thus the in vitro enzymatic reactions confirmed the effect of TesB on CTAL production. The catalytic mechanisms of CHS revealed the formation of thioester intermediates during these reactions (Fig. 1). In naringenin biosynthesis, the thioester intermediate of CHS, compound 1, was not stable and quickly cyclized to form lactone CTAL upon CoA hydrolyzation, whereas CTAL could no longer be converted to naringenin (Fig. 1). Our results suggest that TesB, which catalyzes the hydrolysis of acyl-CoA substrates, could improve CTAL yields. This is likely owing to the release of thioester intermediates 1 from CHS enzymes, which were cleaved by TesB outside of the catalytic pocket, leading to the formation of CTAL, which could no longer be converted.

Previously, the mechanism of byproduct formation in CHS was suggested to involve a lactonization-type ring closure, other than Claisen condensation in the catalytic pocket [17, 36, 37]. Our results indicate that it may also result from the release of catalytic intermediates from the catalytic site. Since compounds **1** was not commercially available, direct thioesterase catalysis was not performed. However, both in vivo and in vitro reactions had supported our prediction that the catalytic intermediate of CHS, compound **1**, was at least partially released to the environment and converted to CTAL byproduct when CoA was hydrolyzed. Therefore, strategies preventing the release of such intermediates should reduce byproduct formation and promote naringenin chalcone production.

The T131A mutant obtained in this study displayed dramatic changes in product and byproduct formation (Fig. 3D and E). To further understand the interactions between compound 1 and CHS wild-type or T131A mutant, molecular docking and 20-ns molecular dynamics simulations were performed for the CHS-compound 1 complexes using YASARA. The average binding energy for compound 1 against CHS wild-type and T131A mutant were -999.2 and -876.0 kJ mol^−1^, respectively (Fig. S4). The calculations indicated that compound 1 showed a weaker interaction with the active site of T131A mutant, comparing with wild-type CHS, thus leading to an easier release of compound 1 from T131A enzyme.

Reaction intermediates are more likely to be released when a protein structure is unstable. Therefore, the FireProt web tool [38] was employed to guide the design of more stable CHS mutants. Six single-point mutations were predicted using an energy-based approach (Table S4) and the corresponding CHS mutants were constructed and applied to the whole-cell biosynthesis of naringenin. Only one mutant—T145M—produced notably higher levels of naringenin, and it simultaneously produced less CTAL than the wild-type enzyme (Fig. 3E). The DynaMut tool revealed that T145M substitution led to the formation of new hydrophobic bonds despite removing weak hydrogen bonds (Fig. S5). The ΔΔG_WT-MT_ was predicted as 0.818 kcal mol^−1^, indicating an increased stability of the T145M protein. Combination of CHS-3–15 mutant and T145M substitution also resulted in further improvement in naringenin and reduction in CTAL synthesis (Fig. S6). These results further indicated that improving CHS stability would reduce the chance of intermediate release and could be a practical way of minimizing byproduct formation. This finding can be potentially expanded to most type III PKSs for reducing byproduct formation.

### Effect of CHIL in naringenin biosynthesis

**Fig. 5:**
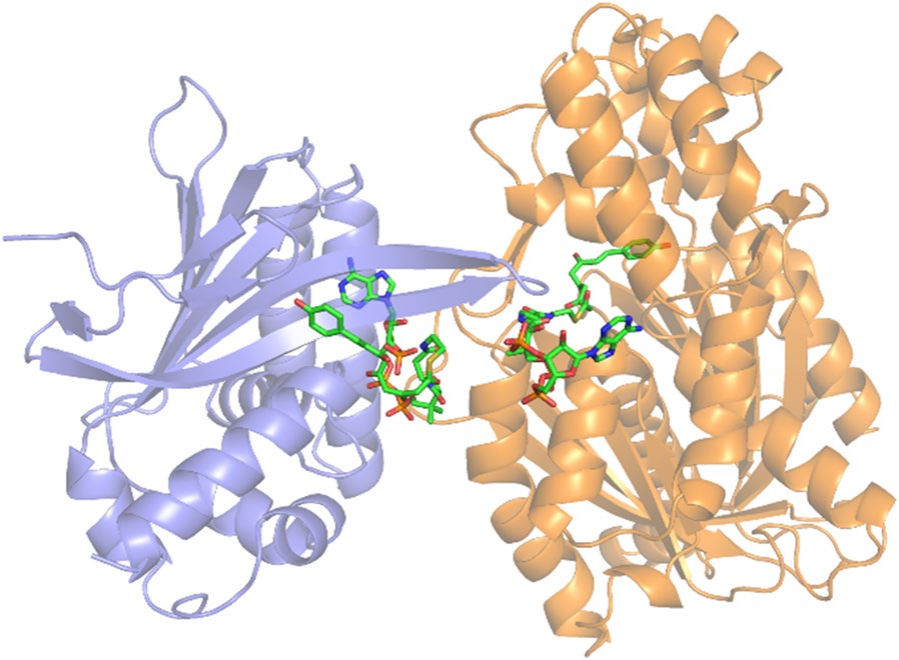
Structure model of CHS–CHIL complex docked with compound **1**. The yellow and purple proteins represent CHS and CHIL, respectively. Compound **1** was docked in both CHS and CHIL

As CHIL has been reported to interact CHS and facilitate naringenin chalcone biosynthesis [21, 22], CHIL from *A. thaliana* was introduced to further improve naringenin production in this study. The results of in vitro reactions confirmed a significant improvement in naringenin production in the presence of CHIL proteins, whereas CTAL levels decreased slightly (Fig. 4B). CHS reactions were then performed in the presence of both CHIL and TesB and we found that, compared to the presence of only CHIL, TesB supplementation further reduced naringenin biosynthesis while improving CTAL formation. However, with the presence of both CHIL and TesB, naringenin biosynthesis remained notably higher than in presence of TesB alone, indicating that CHIL protected the reaction intermediates from degradation by thioesterase (Fig. 4B). In a previous study [22], a binding site of compound 1 was found in the CTAL structure via computational modeling, therefore, we inferred that CHIL bound the compound 1 released from CHS, protecting it from degradation by TesB. The CHIL–CHS complex was modeled and then docked with compound 1 (Fig. 5), and we found that the binding sites of compound 1 in CHS and CHIL were rather close, facilitating the intermolecular transport of this reaction intermediate. Since CHIL interacted with CHS [21, 22], the bound compound 1 could later return to the CHS catalytic pocket again and finally be converted to naringenin chalcone. Therefore, CHIL improved naringenin chalcone synthesis through binding and stabilizing the open-ring configuration of the intermediates during catalysis. Our study provides indirect evidence for the mechanisms of byproduct formation by CHS and CHIL protection for improved naringenin chalcone synthesis, shedding light on developing more efficient whole-cell catalysts for naringenin and downstream flavonoids.

### Development of highly efficient and specific naringenin whole-cell catalyst

The biosynthesis of naringenin and downstream flavonoid compounds is mostly achieved with whole cells. Various thioesterases in a genome can lead to high levels of byproduct formation, lowering the productivity of the final products. Therefore, the inactivation of these thioesterases is necessary for the high-efficiency synthesis of these compounds. As a proof of concept, strain NAR01 was constructed based on strain CUR01, in which five thioesterase genes, *tesB*, *yigI*, *yciA*, *fadM*, and *paaI*, were deleted to reduce CTAL formation. Naringenin biosynthesis was performed with strain NAR01 that harbors plasmids pTrc99a-4CL2M-CHIL/pTrc99a-4CL2M and pYk-CHS, which express the naringenin pathway containing the CHS-3–15 mutant (Fig. 3A), in the presence or absence of CHIL (Fig. 4C and D). The time series of cell growth, product and byproduct formation, and substrate consumption were determined. In the absence of CHIL, naringenin production in strain NAR01 reached 3.24 mM in the flask, with a *E* value of 78.9% (Fig. 4C), while the *E* value was only 50.1% for the same biosynthetic pathway expressed in strain CUR01. Under CHIL expression, naringenin production was further improved to 3.98 ± 0.09 mM (1082 ± 24 mg L^−1^), whereas the accumulation of CTAL was minimized (*E* value = 96.7%) (Fig. 4D). This was the highest production in flasks reported to date [39–41] (Table S5). Therefore, a combination of endogenous thioesterases inactivation and CHIL co-expression finally resulted in an upgraded naringenin whole-cell catalyst with significantly improved product specificity. Combined with the previously reported strategies of engineering intracellular malonyl-CoA availability [42–44], the biosynthetic capability of the whole-cell catalyst was expected to be further improved.

## Conclusions

In conclusion, owing to the inhibition of cell growth by the acyl-CoA starter molecule, a high-throughput screening strategy, coupled with growth-based whole-cell catalysts of type III polyketide naringenin, was developed. This growth selection system has greatly contributed to both enhanced activity and discovery of byproduct formation mechanism in CHS. Our study provided new insights in the catalytic mechanisms of CHS and shed light on engineering heterologous bio-factories to produce high-value type III polyketides at a large scale. Furthermore, the mechanism of this simple and rapid growth-based screening strategy is potentially applicable to the engineering of most type III PKSs, regardless of their final products.

### Supplementary Information

Supplementary file1.

## Data Availability

All data are available either in the main text or in the supplementary information.

## Abbreviations

PKS: Polyketide synthases
CHS: Chalcone synthases
CTAL: *p*-Coumaroyltriacetic acid lactone
SDS-PAGE: Sodium dodecyl sulfate polyacrylamide gel electrophoresis
CHI: Chalcone isomerase
CHIL: CHI-like protein
4CL: *p*-Coumarate:CoA ligase
NTA: Nickel-nitrilotriacetic acid
HPLC: High-performance liquid chromatography
CoA: Coenzyme A
