# Supplementary material for: Designing a highly efficient type III polyketide whole-cell catalyst with minimized byproduct formation
Source: Biotechnol Biofuels Bioprod. 2024 Jul 3;17:93. doi: 10.1186/s13068-024-02545-x (PMC11223281; doi:10.1186/s13068-024-02545-x)
Supplement: Supplementary file 1 — Supplementary file1. [file 13068_2024_2545_MOESM1_ESM.pdf]

Supporting Information

Designing a highly efficient type III polyketide whole-cell catalyst with minimized byproduct formation

La Xiang,[1,3]† Xuanxuan Zhang,[1,3,4]† Yanyan Lei,[1,3,4]† Jieyuan Wu,[1,3,4]† Guangru Yan,[1,3,4] Wei Chen,[1,4] Shizhong Li,[1,3,4] Wenzhao Wang,[5] Jian-Ming Jin,[2,]* Chaoning Liang,[1,3]* Shuang-Yan Tang[1,3]*

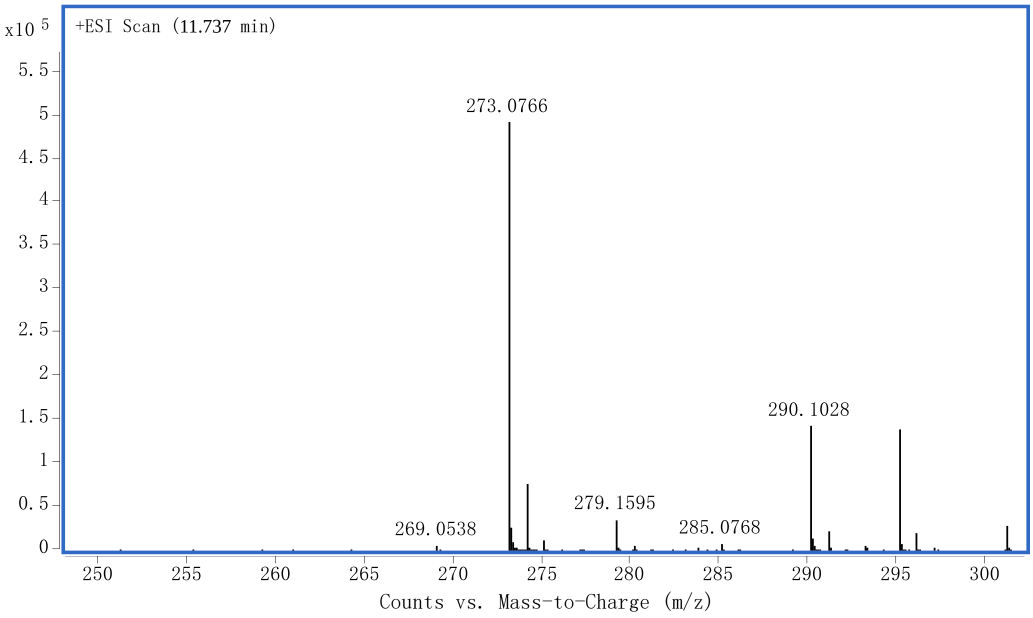

Fig. S1 The mass spectra of CTAL

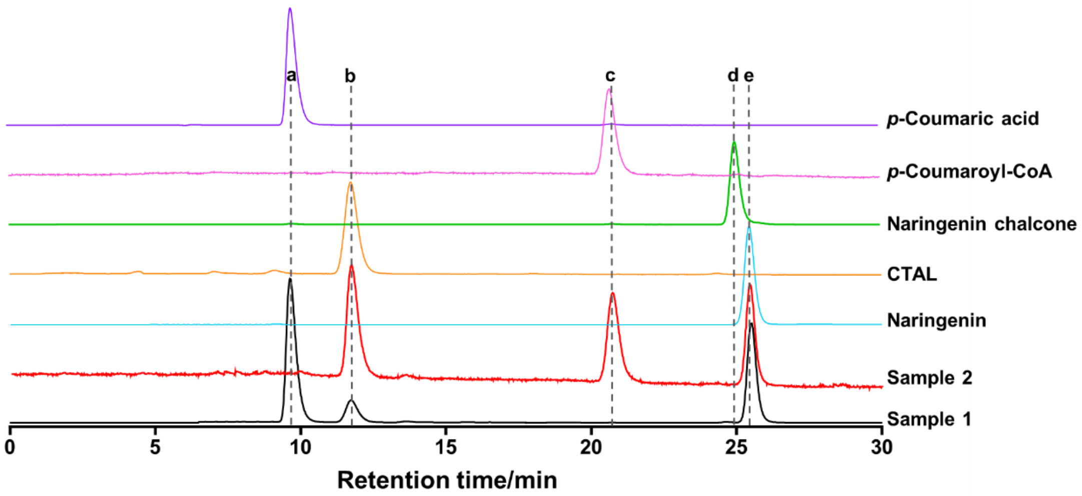

Fig. S2 HPLC chromatograph of the products from strain CUR01 harboring naringenin biosynthetic pathway supplemented with *p*-coumaric acid (Sample 1) or from catalysis of purified CHS using *p*-coumaroyl-CoA and malonyl-CoA as substrates (Sample 2). Retention time of *p*-coumaric acid (**a**), CTAL (**b**), *p*-coumaroyl-CoA (**c**), naringenin chalcone (**d**), and naringenin (**e**) was 9.640, 11.737, 20.674, 24.956, and 25.804 min, respectively

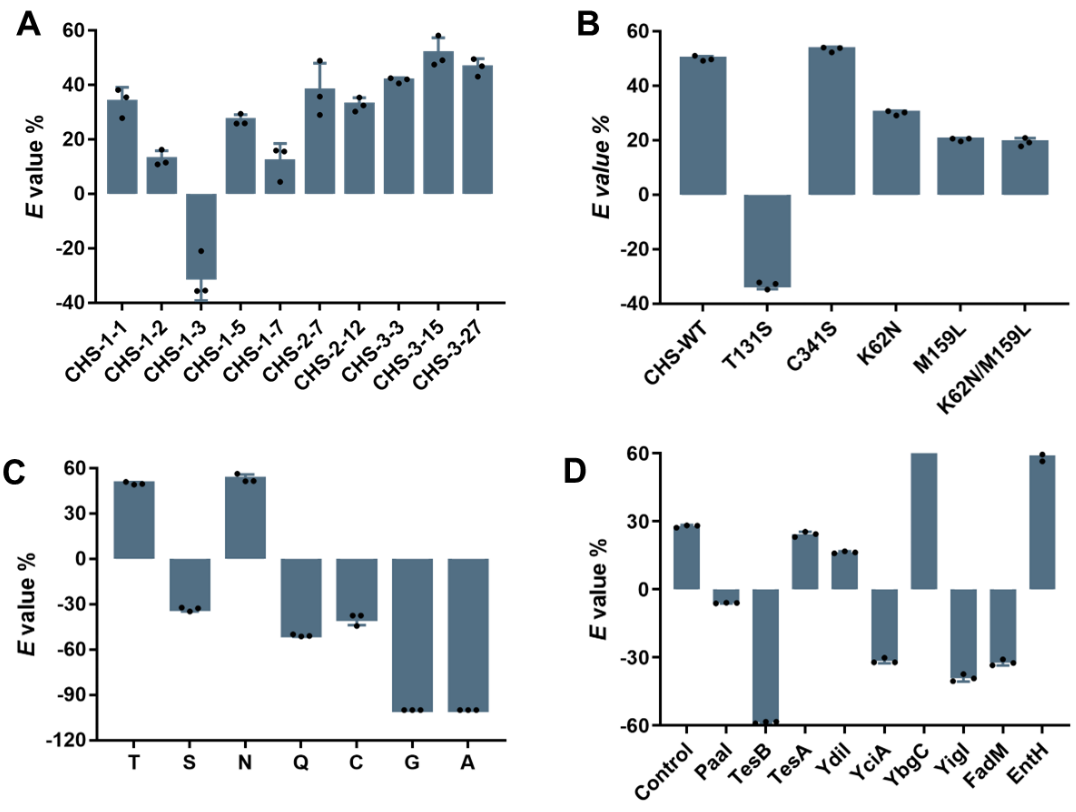

Fig. S3 *E* values of strain CUR01 harboring the naringenin biosynthetic pathway expressing (**A**) CHS mutants selected from three rounds of random mutagenesis; (**B**) the indicated CHS mutants; (**C**) CHS with T131 saturated mutated; or (**D**) wild-type CHS in the presence of selected thioesterases

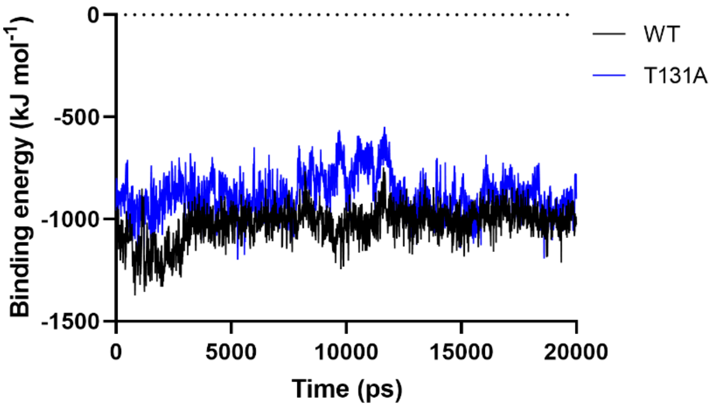

Fig. S4 Molecular dynamics simulations of interactions between compound **1** and wild-type CHS (black) or T131A mutant (blue) in the complexes

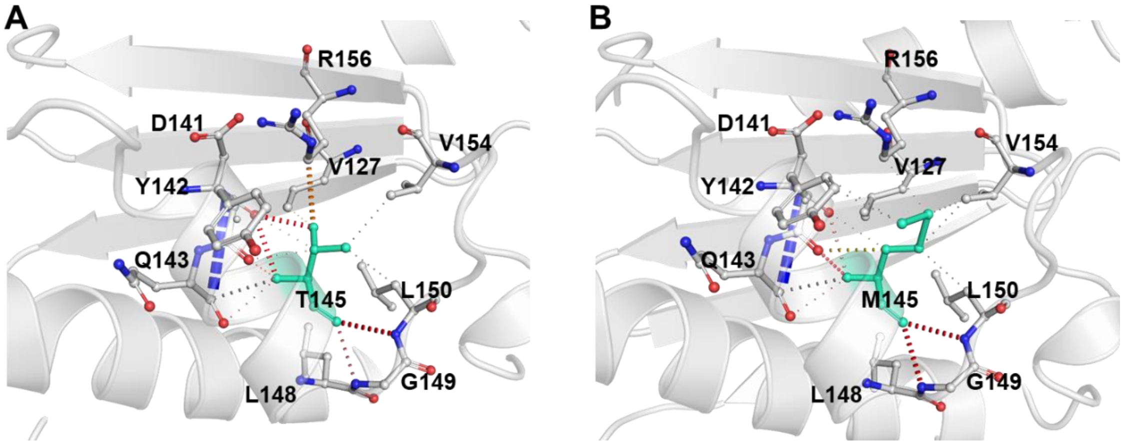

Fig. S5 The interactions between T145 (**A**) or M145 (**B**) and other residues of CHS calculated by DynaMut webserver

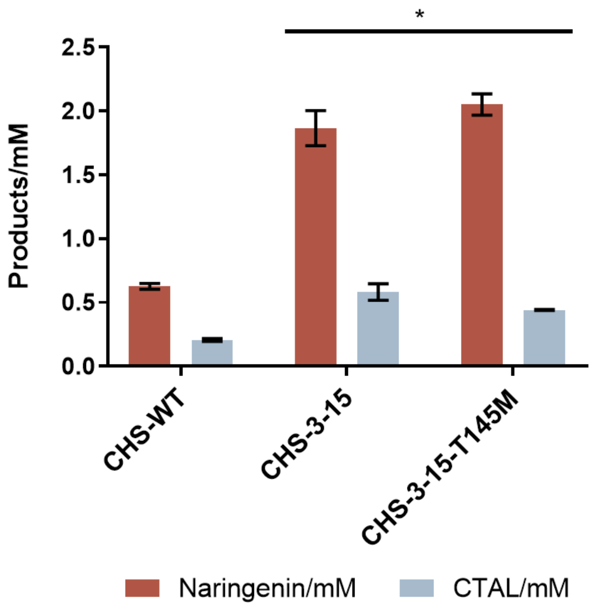

Fig. S6 Naringenin and CTAL productions from strain CUR01 harboring the CHS wild type, mutants CHS-3-15 and CHS-3-15-T145M

Table S1 Strains and plasmids used in this study

Strain	Description	Origin
MC1061	F^- *araD139* Δ(*ara-leu*)*7696 galE15 galK16* Δ(*lac*)*X74 rpsL* (Str^R) *hsdR2* (*rK*$^-$ *mK*$^+$) *mcrA mcrB1*	ATCC 53338
BW25113	F^- Δ(*araD-araB*)567 *lacZ4787*(*del*)::rrnB-3 LAM$^-$ *rph-1* Δ(*rhaD-rhaB*)*568 hsdR514*	[1]
CUR01	BW25113 Δ*poxB*::*acs* Δ*adhE* Δ*fabF*	[2]
NAR01	CUR01 Δ*tesB* Δ*yigI* Δ*yciA* Δ*fadM* Δ*paaI*	this study
**Plasmid**	**Description**	**Origin**
pTrc99a	pBR322-derived vector, P_{trc} promoter. Amp^r	Sangon, Shanghai, China
pTrc99a-4AT	Gene encoding 4CL1 mutant (4AT) under the control of P_{trc} promoter. Amp^r	this study
pTrc99a-4CL2M	Gene encoding 4CL2 mutant (4CL2M) under the control of P_{trc} promoter. Amp^r	this study
pGAP-CHS-4CL	Gene encoding CHS and 4CL under the control of P_{GAP} promoter. Kan^r	[3]
pYk-CHS	Gene encoding CHS under the control of P_{BAD} promoter. Kan^r	this study
pET28a	T7 pET expression plasmid vector. Kan^r	Novagen
pET28a-CHS	Gene encoding CHS under the control of T7 promoter. Kan^r	this study
pET28a-TesB	Gene encoding TesB under the control of T7 promoter. Kan^r	this study
pET28a-CHIL	Gene encoding CHIL under the control of T7 promoter. Kan^r	this study
pTrc99a-4CL2M-CHIL	Gene encoding 4AT and CHIL under the control of P_{trc} promoter. Amp^r	this study
pCA24N-ck	T5lac promoter, Rep Origin, Cm^r	[4]
pCA24N-TesA	Gene *tesA* under the control of T5lac promoter. Cm^r	
pCA24N-TesB	Gene *tesB* under the control of T5lac promoter. Cm^r	
pCA24N-EntH	Gene *entH* under the control of T5lac promoter. Cm^r	
pCA24N-YbgC	Gene *ybgC* under the control of T5lac promoter. Cm^r	
pCA24N-PaaI	Gene *paaI* under the control of T5lac promoter. Cm^r	[5]
pCA24N-YciA	Gene *yciA* under the control of T5lac promoter. Cm^r	
pCA24N-FadM	Gene *fadM* under the control of T5lac promoter. Cm^r	
pCA24N-YigI	Gene *yigI* under the control of T5lac promoter. Cm^r	
pCA24N-YdiI	Gene *ydiI* under the control of T5lac promoter. Cm^r	

Table S2 Primers used in this study

Primers	Sequence
pYk-For	CCACCAGCCATGTGAATTAAGAATTCGGTGAGCTCGGTCT
pYk-Rev	CCCATGGTGGTGGTCGGCGCCATAAGCTTAATTCCTCCTG
CHS-pY-For	TAACAGGAGGAATTAAGCTTATGGTTACGGTGGAAGAAT
CHS-pY-Rev	CCGAGCTCACCGAATTCTTAGGTAGCCACACTATGCAG
pY-CHS-For	ATTCTTCCACCGTAACCATAAGCTTAATTCCTCCTGTTA
pY-CHS-Rev	ATAGTGTGGCTACCTAAGAATTCGGTGAGCTCGGTC
CHS-G-For	CAAATAGCTAGGAGGAACCATATGGTTACGGTGGAAGAATACCG
CHS-G-Rev	CGGCACCAGCTGCAGACCTTTTAGGTAGCCACACTATGCAGA
28a-CHS-G-For	CGGCAGCCATATGGCTAGCATGGTTACGGTGGAAGAATAC
28a-CHS-G-Rev	CTTGTCGACGGAGCTCGAATTCTTAGGTAGCCACACTATGCAGAA
pET28a-fwd	TTCTGCATAGTGTGGCTACCTAAGAATTCGAGCTCCGTCGACAAG
pET28a-rev	GTATTCTTCCACCGTAACCATGCTAGCCATATGGCTGCCG
tesB-GB-For	CGGCAGCCATATGGCTAGCATGAGTCAGGCGCTAAAAAAT
tesB-GB-Rev	TGTCGACGGAGCTCGAATTCTTAATTGTGATTACGCATCA
pET-tesB-For	TGATGCGTAATCACAATTAAGAATTCGAGCTCCGTCGACA
pET-tesB-Rev	ATTTTTTAGCGCCTGACTCATGCTAGCCATATGGCTGCCG
CHIL-For	CTGAGCAAATAAGTCGACAAGCTTGCGGCCGC
CHIL-Rev	ATATCATCAGAACCGTGGTGGTGATGATGATGATGATGGCTG
pET28a-CHIL-For	ACCACGGTTCTGATGATATCCCAACGACCGAAAACCTGTATTTTCAGG
pET28a-CHIL-Rev	GCTTGTCGACTTATTTGCTCAGTTCTGCGC
CHIL-gibson-For	CAAATGGATTAATGAACTAGTCACACAGGAAACAGACCATGGAAAACAACATGGTTAT
CHIL-gibson-Rev	TCTCATCCGCCAAAACAGCCTTATTTGCTCAGTTCTGCGC
4CL2M-G-For	GCGCAGAACTGAGCAAATAAGGCTGTTTTGGCGGATGAGA
4CL2M-G-Rev	ATAACCATGTTGTTTTCCATGGTCTGTTTCCTGTGTGACTAGTTCATTAATCCATTTG
4AT-G-For	CACACAGGAAACAGACCATGGCGCCACAAGAACAAGC
4AT-G-Rev	CATCCGCCAAAACAGCCTCACAATCCATTTGCTAGTTTTG
pTrc99a-G-For	AGGCTGTTTTGGCGGATGAG
pTrc99a-G-Rev	GGTCTGTTTCCTGTGTGAAATTG
CHIL-Rev	TTATTTGCTCAGTTCTGCGC
CHS-EP-For	ATGGTTACGGTGGAAGAAT
CHS-EP-Rev	TTAGGTAGCCACACTATGC
CHS-T131A-For	ACCCATCTGGTCTTTTGCGCTACGTCGGGTGTGG

Note: XXX represents 19 codons for site-directed mutagenesis of each residue.

Table S3 Genes encoding functional proteins from *E. coli* used in this study

Genes	Function of encoding proteins
yciA	acyl-CoA thioesterase
tesB	acyl-CoA thioesterase II
tesA	multifunctional acyl-CoA thioesterase I and protease I and lysophospholipase
ybgC	esterase/thioesterase
entH	thioesterase in enterobactin biosynthesis
yigI	acyl-CoA thioesterase involved in thioesterase-dependent β-oxidation of fatty acids
fadM	long-chain acyl-CoA thioesterase
ydiI	1,4-dihydroxy-2-naphthoyl-CoA thioesterase enzyme in the biosynthesis of menaquinone
paaI	phenylacetyl-CoA thioesterase
hyfA	hydrogenase 4 component A

Table S4 Change of Gibbs free energy in different mutants calculated by FireProt web server

**Mutation**	**Change of Gibbs free energy (kcal mol^{-1})**	**Position**
V2M	-1.16	loop
C84R	-3.16	loop
Q119M	-1.93	loop
Q143Y	-1.59	α-helix
T145M	-1.36	α-helix
G359P	-2.28	loop

Table S5 Microbial mono-culture for the production of naringenin in the recent years

Substrate	Host Strain	Titer (mg L^{-1})	Fermentation	Reference
p-Coumaric acid	*Corynebacterium glutamicum*	35	flask	[6]
Tyrosine	*Saccharomyces cerevisiae*	90		[7]
Tyrosine	*E. coli*	191.9	flask	[8]
Glucose	*Yarrowia lipolytica*	71.2		[9]
Glucose	*Y. lipolytica*	252.4	flask	[10]
p-Coumaric acid	*S. cerevisiae*	648.63	fed-batch	[11]
Glycerol	*E. coli*	484	flask	[12]
Glucose	*E. coli*	588	bioreactor	[13]
Glucose	*E. coli*	523.7	bioreactor	[14]
Glucose, tyrosine	*E. coli*	1073.8	bioreactor	[15]
p-Coumaric acid	*E. coli*	1082	flask	This study
Glycerol	*E. coli*	272.2	fed-batch	[16]

Reference

1. Datsenko KA, Wanner BL. One-step inactivation of chromosomal genes in *Escherichia coli* K-12 using PCR products. Proc Natl Acad Sci U S A. 2000; 97(12): 6640-5.
2. Wu J, Chen W, Zhang Y, Zhang X, Jin JM, Tang SY. Metabolic engineering for improved curcumin biosynthesis in *Escherichia coli*. J Agri Food Chem. 2020; 68(39): 10772-9.
3. Xiong D, Lu S, Wu J, Liang C, Wang W, Wang W, et al. Improving key enzyme activity in phenylpropanoid pathway with a designed biosensor. Metab Eng. 2017; 40: 115-23.
4. Zhang X, He Y, Wu Z, Liu G, Tao Y, Jin JM, et al. Whole-cell biosensors aid exploration of vanillin transmembrane transport. J Agri Food Chem. 2021; 69(10): 3114-23.
5. Kitagawa M, Ara T, Arifuzzaman M, Ioka-Nakamichi T, Inamoto E, Toyonaga H, et al. Complete set of ORF clones of *Escherichia coli* ASKA library (a complete set of *E. coli* K-12 ORF archive): unique resources for biological research. DNA research : an international journal for rapid publication of reports on genes and genomes. 2005; 12(5): 291-9.
6. Kallscheuer N, Vogt M, Stenzel A, Gaetgens J, Bott M, Marienhagen J. Construction of a *Corynebacterium glutamicum* platform strain for the production of stilbenes and (2*S*)-flavanones. Metab Eng. 2016; 38: 47-55.
7. Lyu X, Ng KR, Lee JL, Mark R, Chen WN. Enhancement of naringenin biosynthesis from tyrosine by metabolic engineering of *Saccharomyces cerevisiae*. J Agric Food Chem. 2017; 65(31): 6638-46.
8. Zhou S, Lyu Y, Li H, Koffas MAG, Zhou J. Fine-tuning the (2S)-naringenin synthetic pathway using an iterative high-throughput balancing strategy. Biotechnol Bioeng. 2019; 116(6): 1392-404.
9. Lv Y, Edwards H, Zhou J, Xu P. Combining 26s rDNA and the Cre-loxP system for iterative gene integration and efficient marker curation in *Yarrowia lipolytica*. ACS Syn Biol. 2019; 8(3): 568-76.
10. Lv Y, Marsafari M, Koffas M, Zhou J, Xu P. Optimizing oleaginous yeast cell factories for flavonoids and hydroxylated flavonoids biosynthesis. ACS Syn Biol. 2019; 8(11): 2514-23.
11. Gao S, Lyu Y, Zeng W, Du G, Zhou J, Chen J. Efficient biosynthesis of (2*S*)-naringenin from *p*-coumaric acid in *Saccharomyces cerevisiae*. J Agric Food Chem. 2020; 68(4): 1015-21.
12. Dunstan MS, Robinson CJ, Jervis AJ, Yan C, Carbonell P, Hollywood KA, et al. Engineering *Escherichia coli* towards *de novo* production of gatekeeper (2*S*)-flavanones: naringenin, pinocembrin, eriodictyol and homoeriodictyol. Syn Biol (Oxford, England). 2020; 5(1): ysaa012.
13. Zhou S, Hao T, Zhou J. Fermentation and metabolic pathway optimization to *de novo* synthesize (2*S*)-naringenin in *Escherichia coli*. J Microbiol Biotechnol. 2020; 30(10): 1574-82.
14. Zhou S, Yuan S-F, Nair PH, Alper HS, Deng Y, Zhou J. Development of a growth coupled and multi-layered dynamic regulation network balancing malonyl-CoA node to enhance (2S)-naringenin biosynthesis in *Escherichia coli*. Metab Eng. 2021; 67: 41-52.
15. Wu J, Zhou L, Duan X, Peng H, Liu S, Zhuang Q, et al. Applied evolution: Dual dynamic regulations-based approaches in engineering intracellular malonyl-CoA availability. Metab Eng. 2021; 67: 403-16.
16. Jiang T, Li C, Zou Y, Zhang J, Gan Q, Yan Y. Establishing an autonomous cascaded artificial dynamic (AutoCAD) regulation system for improved pathway performance. Metab Eng. 2022; 74: 1-10.
